# Cognitive and behavioral involvement in ALS has been known for more than a century

**DOI:** 10.1007/s10072-022-06340-0

**Published:** 2022-09-02

**Authors:** Stefano Zago, Lorenzo Lorusso, Edoardo N. Aiello, Martino Ugolini, Barbara Poletti, Nicola Ticozzi, Vincenzo Silani

**Affiliations:** 1 U.O.C. Neurologia, Fondazione IRCCS Ospedale Maggiore Policlinico, Università degli Studi di Milano, Milan, Italy; 2 U.O.C., Neurologia & Stroke Unit, A.S.S.T. Lecco, Merate, LC Italy; 3 PhD Program in Neuroscience, School of Medicine and Surgery, University of Milano-Bicocca, Monza, Italy; 4 IRCCS Istituto Auxologico Italiano, Department of Neurology and Laboratory of Neuroscience, Milan, Italy; 5 Center for Integrative Genomics, University of Lausanne, Lausanne, Switzerland; 6 Department of Pathophysiology and Transplantation, “Dino Ferrari Center”, Università degli Studi di Milano, Milan, Italy

**Keywords:** Amyotrophic lateral sclerosis, Frontotemporal degeneration, Neuropsychology

## Abstract

**Background:**

Among clinicians and researchers, it is common knowledge that, in ALS, cognitive and behavioral involvement within the spectrum of frontotemporal degenerations (FTDs) begun to be regarded as a fact in the late 1990s of the twentieth century. By contrast, a considerable body of evidence on cognitive/behavioral changes in ALS can be traced in the literature dating from the late nineteenth century.

**Methods:**

Worldwide reports on cognitive/behavioral involvement in ALS dating from 1886 to 1981 were retrieved thanks to Biblioteca di Area Medica “Adolfo Ferrate,” Sistema Bibliotecario di Ateneo, University of Pavia, Pavia, Italy and qualitatively synthetized.

**Results:**

One-hundred and seventy-four cases of ALS with co-occurring FTD-like cognitive/behavioral changes, described in Europe, America, and Asia, were detected. Neuropsychological phenotypes were consistent with the revised Strong et al.’s consensus criteria. Clinical observations were not infrequently supported by histopathological, post-mortem verifications of extra-motor, cortical/sub-cortical alterations, as well as by in vivo instrumental exams—i.e., assessments of brain morphology/physiology and psychometric testing. In this regard, as earlier as 1907, the notion of motor and cognitive/behavioral features in ALS yielding from the same underlying pathology was acknowledged. Hereditary occurrences of ALS with cognitive/behavioral dysfunctions were reported, as well as familial associations with ALS-unrelated brain disorders. Neuropsychological symptoms often occurred before motor ones. Bulbar involvement was at times acknowledged as a risk factor for cognitive/behavioral changes in ALS.

**Discussion:**

Historical observations herewith delivered can be regarded as the antecedents of current knowledge on cognitive/behavioral impairment in the ALS-FTD spectrum.

**Supplementary Information:**

The online version contains supplementary material available at 10.1007/s10072-022-06340-0.

## Introduction

In spite of the seminal description of amyotrophic lateral sclerosis (ALS) delivered in 1869 by Jean-Martin Charcot and Alix Joffroy, who introduced the notion of “the mind [being] unaffected in ALS” [119], it took no longer than 23 years for Pierre Marie, one of the most distinguished protégés of Charcot himself, to firmly reply “yes” to his own question whether “mental functions are altered over the course of [ALS]” (p. 470) [65].

It is thereupon surprising that, among clinicians and researchers, the pathophysiological, genetic, and phenotypic link between frontotemporal degeneration (FTD) and ALS (i.e., the ALS-FTD spectrum) [18, 124] begun to be regarded as a fact only between the late 1990s and early 2000s of the twenty-first century.

Indeed, the scientific and clinical community has been provided with a nosographic system for FTD-spectrum disorders in these patients not earlier than 2009 [99, 100]. El Escorial diagnostic criteria, in fact, still under-addressed cognitive and behavioral features in ALS [16].

By contrast, within the present historical review, it is demonstrated that the recognition of extra-motor, FTD-spectrum disorders in ALS dates back at least 130 years. Herewith, European, American, and Asian reports starting from 1882 and suggestive of cognitive/behavioral changes in 174 ALS patients are presented. Such records were retrieved thanks to Biblioteca di Area Medica “Adolfo Ferrate,” Sistema Bibliotecario di Ateneo, University of Pavia, Pavia, Italy, and qualitatively synthetized. Records herewith described have been searched for up to 1981, as this being the date of publication of the first, pioneering review by Hudson [54] that actually acknowledged the association between ALS and cognitive/behavioral changes. However, Hudson’s [54] review was not exhaustive of all the records preceding 1981—which are, indeed, by far less known by the modern scientific community, and were thus intended to be brought to the light within this work.

## Early clinical observations

Within the 10 years following the 1892 acknowledgment by Marie of “[mental] disturbances [being] not only highly frequent in [ALS] but also typical [of it]” (p. 470) [65], several authors worldwide begun reporting emotional lability, gelastic/dacrystic episodes, anosognosia as well as disinhibited and psychotic traits in ALS patients [80, 95, 115]. Notably, Marie [65] himself had already listed such alterations among the most characteristic features of their neuropsychological profile—in striking overlap with the current knowledge on dysexecutive behavioral phenotypes within the ALS-FTD spectrum [83].

As to cognition, early semeiotic reports described, besides a non-specific decrease in global efficiency [80, 39, 49, 95], memory deficits [105], as well as oral and written language disturbances [115, 56]—which are cognitive features currently acknowledged as typical of the ALS-FTD spectrum [83].

**Fig. 1:**
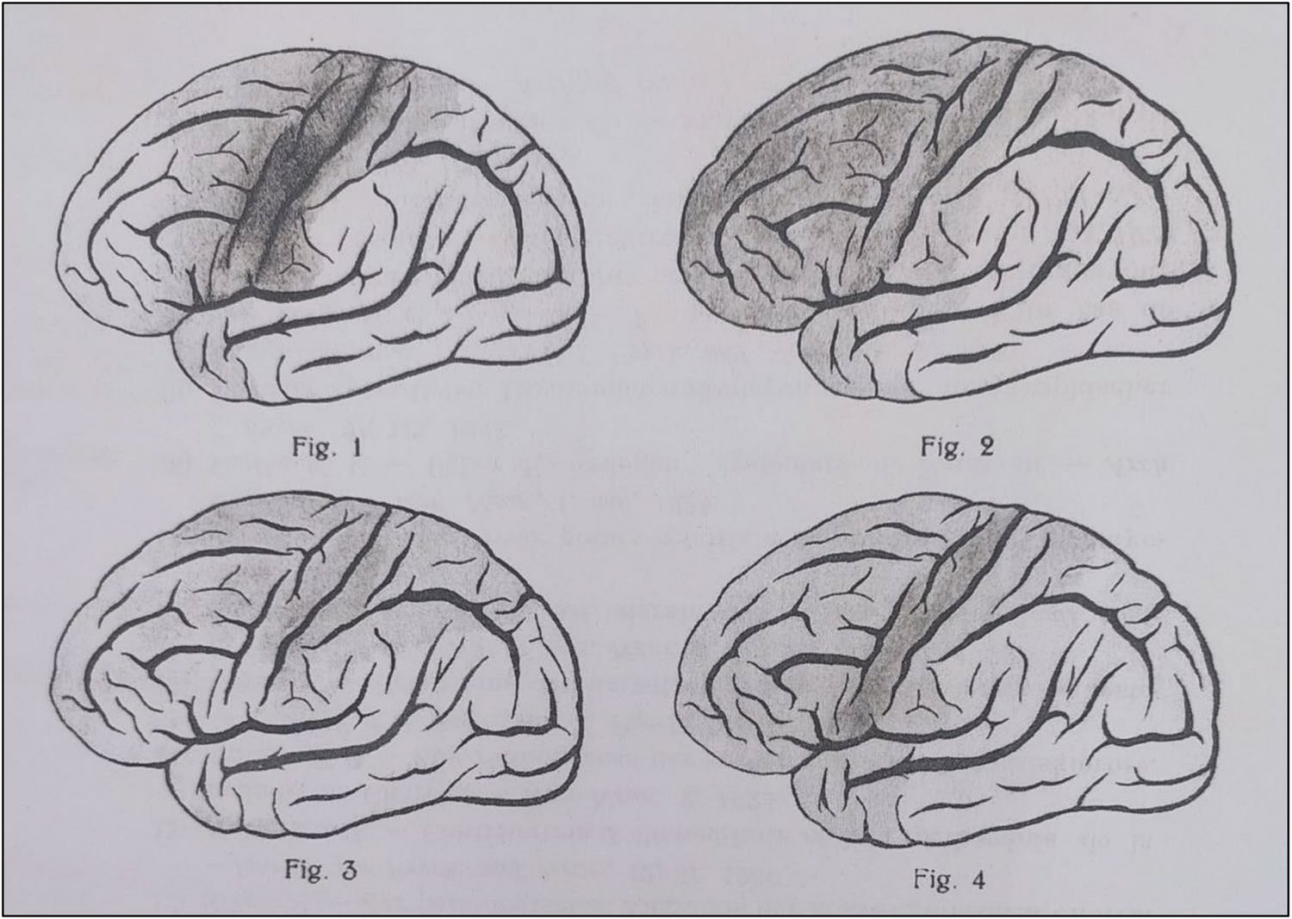
Post-mortem evidence of neuropathology within the frontotemporal cortex in a case series of ALS patients described by Bartoloni and D’Angelo [9]. Notes. ALS, amyotrophic lateral sclerosis

A turning point for the full recognition of neuropsychological involvement in ALS occurred after 1905, with Cullerre, in 1906, being the first to explicitly address it by entitling his report “*Trouble mentales dans le sclerose laterale amyotrophique*”—i.e., “Mental disturbances in [ALS]” [29]. Notably, the modern parallel of such a title dates back not earlier than 2003, with Lomen-Hoerth et al. [63] posing the question “*Are amyotrophic lateral sclerosis patients cognitively normal?*.” One year after Cullerre’s [29] report, Fragnito [37] forwarded the pioneeristic notion of motor and cognitive/behavioral features in ALS not being unrelated, but rather yielding from the same underlying pathology spreading to extra-motor, frontotemporal cortices. Notably, such a hypothesis subsequently gained greater support over the second-to-sixth decades of the twentieth century: for instance, Bartoloni and D’Angelo [9] delivered, exactly 40 years after Fragnito [37], strikingly clear post-mortem evidence of frontotemporal cortex involvement in ALS (Fig. 1).

As to ante litteram stances, Fragnito [37] and Gentile [42] also noticed that neuropsychological disorders in ALS are heterogeneous in severity, somehow anticipating the current notion of a continuum of these features, ranging from sub-clinical/mild deficits (i.e., the current nosological entities of ALS-cognitive/-behavioral/-cognitive and behavioral impairment, ALSci/bi/cbi) [99] to a full-blown dementing state.

Interestingly, Cullerre [29] further described sitophobia (i.e., aversive behaviors towards food) and anosognosia in ALS patients—both features being consistent with the current knowledge of FTD-spectrum dysfunction in ALS [83].

In respect to illness awareness, it is of note that several authors reported mild-to-absent [91], moderate [92], or severe [39] loss of insight into either motor disabilities or neuropsychological changes—this being consistent with the now recognized continuum of severity of anosognosic features in ALS [83]. Relevantly, Resegotti [92] also underlined the detrimental impact of anosognosic features on adherence to and compliance with treatments—which is a renowned issue in the clinical management of ALS patients [55].

## Twentieth-century clinical reports

Possibly due to the progressive acknowledgment of the aforementioned notions, starting from 1907, worldwide reports of neuropsychological impairment in ALS patients both increased in frequency and improved as to semeiotic accuracy (Table 1).

**Table 1:** Summary of extracted outcomes

Author(s), year	*N*	Age, sex	NPs onset	DD	Bulbar signs	EPD	Cognitive deficits	Behavioral alterations	Post-mortem histological findings	In vivo exams	Familial elements
Oppenheim and Siemerling 1886 [80]	5	NA	–	NA	All: +	–	All: cognitive efficiency	All: PBA	All: widespread atrophy	NA	–
Marie 1887 [66]	1	31, M	–	23 mo	+	–	Cognitive efficiency	PBA	NA	NA	–
Tranquilli 1890 [105]	1	45, M	–	3 y	+	–	Memory	PBA	NA		–
Marie 1892 [65]	2	NA	+	P1: 10 y. P2: 13 y	NA	NA	P1 and P2: cognitive efficiency	P1 and P2: psychosis	NA	NA	–
Watanabe 1893 [115]	1	41, M	–	NA	+	–	Language	Disinhibition	NA	NA	–
Sarbó 1898 [95]	1	55, M	+	2 y	+	–	Cognitive efficiency	PBA	Widespread atrophy	NA	–
Franceschi 1902 [39]	1	56, M	+	1 y	+	–	–	Depression Anosognosia Disinhibition PBA	F	NA	–
Haenel 1903 [49]	1	45, F	–	5 mo	+	–	–	Disinhibition	Widespread atrophy	NA	–
Mass 1904 [68]	2	P1: 29, M P2: 26, F	–	NA	+	+	P2: cognitive efficiency	P1 and P2: disinhibition	NA	NA	+
Raymond and Cestan 1905 [91]	1	36, M	NA	28 mo	+	–	–	Depression Anosognosia	NA	NA	–
Cullerre 1906 [29]	5	P1: 47 M P2: 44 M P3: 50, M P4: 2 M P5: 49, M	P1, P2, P4, P5: +	P1: 1 y P2, P3, P5: NA; P4: 3 y	P1, P2, P3, P5: +	–	P1, P3: – P2: memory, cognitive efficiency P4: cognitive efficiency P5: cognitive efficiency	P1: psychosis, depression, eating behavior, anosognosia P2: depression, psychosis P3: depressionP4: psychosis, PBA P5: psychosis	NA	NA	P1: + (epilepsy)
Resegotti 1907 [92]	1	40, M	–	2 y	+	–	Executive functioning	PBA	Widespread atrophy	NA	–
Fragnito 1907 [37]	3	P1: 53, M P2: 44, M P3: 41, M	–	P1: 1 y P2: 3 y P3: NA	P1: + P2: + P3:	P1: – P2: P3:	P1: orientation, memory P2: executive functioning, memory P3: disorientation, memory, executive functioning	P1: depression, apathy P2: apathy, anosognosia P3: apathy, anosognosia, psychosis, depression	NA	NA	P2: + (epilepsy) P3: + (stroke)
Gentile 1909 [42]	2	P1: 54, M P2: 20, M	–	NA	P1 and P2: +	–	P1 and P2: cognitive efficiency, executive functioning, memory	P1: depression	NA	NA	–
Fernàndez 1908 [35]	1	51, M	–	NA	+	–	Executive functioning	Depression	NA	NA	+ (stroke)
Astwazaturow 1911 [6]	1	50, M	–	12 y	–	–	–	Depression Psychosis	NA	NA	–
Barrett 1913 [8]	1	35, F	+	5 y	+	–	Cognitive efficiency, orientation Language	Disinhibition Depression PBA	Widespread atrophy; AD	NA	–
Marchand and Dupouy 1914 [64]	1	44, M	+	NA	–	–	Cognitive efficiency, memory	Depression	NA	NA	–
Martini 1916 [67]	3	P1: 26 M P2: 39 M	–	NA	–	P1 and P2: + P3: –	P1: executive functioning P2: – P3: executive functioning, cognitive efficiency	P1: PBA P2: PBA, depression P3: PBA, depression, disinhibition	NA	NA	–
Fragola 1918 [38]	1	57, M	+	2 y	+	+	Memory	Psychosis Depression Anxiety PBA	F-P (L > R)	NA	–
Büscher 1922 [19]	1	42, M	+	16 mo	+	+	Memory	Disinhibition	F	NA	–
Gerber and Naville 1921 [44]	1	43, M	–	3 y	+	–	–	Depression, disinhibition, anosognosia	F	NA	–
Tscherning 1921 [108]	1	30, M	–	NA	–	–	–	Psychosis	NA	NA	+
Matzdorff 1925 [69]	1	38, M	–	4 y	+	+	–	–	P1: F	NA	–
Tretiakoff and Amorin 1924 [106]	1	25, F	+	1 y	+	–	Memory Executive functioning	Apathy	–	NA	–
Van Bogaert 1925 [109]	10	P1: 47, F P2: 68, F P3: 62, F P4: 64, M P5: 34, M P6: 42, F P7: 63, F P8: 34, M P9: 57, F P10: 52, M	P3 and P4: + M P6 and P7: +	NA	P2, P3, P4, P8, P9, P19: +	–	P1: – P2: – P3: – P4: cognitive efficiency P5: memory, cognitive efficiency P6: – P7: cognitive efficiency P8: memory, cognitive efficiency P9: memory, orientation P10: –	P1: depression, disinhibition P2: depression, apathy P3: depression P4: depression, disinhibition P5: disinhibition, apathy P6: disinhibition P7: depression, apathy P8: disinhibition, psychosis P9: apathy, disinhibition P10: depression	NA	NA	–
Westphal 1925 [118]	2	P1: 46, M P2: 43, M	–	NA	+	+	–	Psychosis	NA	NA	–
Meyer 1929 [72]	1	55, M	+	9 mo	+	–	Memory	Disinhibition Apathy OCD psychosis	F; sub-cortical	NA	–
Ottonello 1929 [81]	5	P1: 46, F P2: 44, M P3: 30, M P4: 40, M P5: 50, F	P2: +	P1, P2, P3: NA P4: 2 y P5: 2 ½ y	P1, P3, P4, P5: +	All: –	All: –	P1: depression, PBA P2: depression P3: PBA P4: PBA P5: disinhibition; psychosis	NA	NA	P5: + (stroke)
Ziegler 1930 [125]	2	51, M 59, F	P2: +	P1: 1 y P2: 3 y	All: +	–	P1: cognitive efficiency P2: cognitive efficiency, memory	P1: disinhibition, PBA P2: psychosis	NA	NA	–
Munch-Petersen 1931 [77]	3	P1: 20, F P2: 20, F P3: 30, F	–	NA	P1: + P2 and P3: –	–	P2: cognitive efficiency	P1: disinhibition P3: psychosis	NA	NA	+
Von Braunmùhl 1932 [113]	1	40, M	+	2 y	–	–	Executive functioning, cognitive efficiency	Disinhibition Psychosis	T; Pick’s	NA	–
Wechsler and Davidson 1932 [116]	1	38, M	+	2.2 y	+	–	Memory Attention Language	Psychosis Depression Apathy PBA	F	NA	–
Teichmann 1935 [103]	1	43, M	+	16 mo	–	–	–	Depression, psychosis	Sub-cortical; cerebellar	NA	–
Gozzano 1936 [48]	2	P1: 42, M P1: 40, M	+ –	3 y 2 y	+ –	– –	P1: + P2: memory	P1: PBA, apathy P2: anosognosia, apathy	F	NA	P2: + (psychiatric)
Miskolczy and Csermely 1939 [76]	1	64, M	+	5 y	–	–	Language Cognitive efficiency	Eating behavior	Pick’s L > R F-T	EEG: F-T	+ (psychiatric)
Androp 1940 [5]	1	55, M	–	6 y	–	–	Cognitive efficiency	Psychosis	F-P–T	NA	–
Friedrich 1940 [41]	1	35, M	–	3 y	–	–	Memory	Depression, disinhibition	F; Pick’s	NA	+
Meller 1940 [71]	1	59, M	+	NA	–	–	–	Psychosis, disinhibition	NA		–
Tirico 1940 [104]	1	42, M	+	NA	+	–	Memory, language, orientation	Psychosis	NA	NA	–
Von Bagh 1941 [112]	30	NA	NA	NA	NA	+	NA	NA	6 pts.: F 11 pts: T 13 pts.: F-T 9 pts: P	NA	–
De Caro 1941 [31]	1	NA, M	+	NA	+	–	Cognitive efficiency, language, visuo-spatial	PBA	F-P (L > R); sub-cortical	NA	–
Raithel 1941 [89]	1	–	+	NA	–	–	–	Psychosis	–	NA	+
Tronconi 1941 [107]	1	58, M	+	NA	+	–	Orientation, memory, attention, language	PBA	F; T (L); sub-cortical; Pick’s	NA	–
Van Reeth et al. 1961 [110]	1	52, M	+	6 y	–	–	Executive functioning, memory Praxis	Eating behavior, disinhibition	F-T (+ T); CC; OFC	PEG: F	–
Bartoloni and D’Angelo 1947 [9]	4	P1: 53, F P2: 52, M P3: 50, M P4: 44, F	P4: +	P1: 3 y P2: NA P3: 6 y P4: 4 y	P1: – P2: + P3: + P4: –	–	P1: – P2: attention P3: attention P4: attention	P1: depression P2: disinhibition P3: depression, anxiety P4: disinhibition; psychosis	P1: F; sub-cortical P2: F-T; sub-cortical P3: F-P; sub-cortical P4: F; sub-cortical	NA	–
Friedlander and Kesert 1948 [40]	1	50, M	+	3 y	+	–	Language, cognitive efficiency, executive functioning	Disinhibition, psychosis	NA	NA	–
Bartoloni 1950 [10]	1	58, M	+	11 y	+	–	Executive functioning, memory	Disinhibition, apathy, anosognosia	NA	NA	–
Robertson 1953 [94]	1	69, F	–	13 mo	+	–	Memory, language, orientation, executive functioning, attention	Disinhibition, apathy, psychosis	F-T	NA	+
Léchelle et al. 1954 [61]	1	44, M	+ M	3 y	+	–	Memory, language	Disinhibition	Pick’s; F	NA	–
Michaux et al. 1955 [73]	2	P1 and P2: 58	All: + M	P1 and P2: 2 y	P1 and P2: +	–	P1: cognitive efficiency, language, praxis, visuo-spatial P2: language; praxis; visuo-spatial; memory	P1: disinhibition	P1: F-P–T (L > R)	P1: EEG and CSF: negative	–
Corsino and Lugaresi 1956 [28]	2	P1: 49, M P2: 53, M	P1: +	P1 and P2: NA	P1 and P2: +	–	–	P1: psychosis, depression P2: apathy, depression, PBA	NA	NA	–
Levi 1958 [62]	1	53, F	–	2	+	–	Cognitive efficiency, attention, memory, executive functioning	Disinhibition, depression, anxiety, psychosis	Widespread atrophy; sub-cortical	CSF: negative	–
Bonaretti 1959 [12]	2	P1: 67, M P2: 66, M	All: +	P1: 3 y P2: NA	P1: +		P1: attention, executive functioning, calculation, orientation, memory, cognitive efficiency P2: memory, orientation, cognitive efficiency	P1: disinhibition, PBA, depression P2: apathy, PBA, depression	NA	NA	–
Campanella and Bigi 1959 [21]	1	60, M	–	20 y	–	–	Attention, executive functioning, memory, cognitive efficiency, praxis	Apathy, anosognosia, disinhibition	NA	NA	+
Delay et al. 1959 [33]	2	P1: 57, F P2: 61, M	–	P1: 2 y P2: 3 y	All: +	–	P1: cognitive efficiency, attention, executive functioning, orientation P2: language	P1: psychosis, disinhibition, PBA P2: psychosis	P1: F-T P2: F, T, P, O	NA	–
Gentili and Volterra 1960 [43]	4	P1: 51, F P2: 63, F P3: 56, F P4: 48, F	All: +	P1, P3: 18 mo P2: 1 y P4: 4 ½ y	All: +	–	P1: attention, language, cognitive efficiency P2: attention, language, orientation, cognitive efficiency P3: cognitive efficiency P4: language, memory, calculation	P1: apathy, disinhibition P2: apathy, disinhibition P3: apathy P4: depression	NA	P1: CSF: negative EEG: widespread abnormalities PEG: widespread atrophy P2: CSF: negative EEG: widespread abnormalities PEG: F P3: EEG: widespread abnormalities PEG: widespread atrophy	–
Hanau 1960 [51]	1	45, M	+	NA	+	–	Memory, attention, cognitive efficiency	Apathy	NA	PEG: F EEG: negative	–
Smith 1960 [98]	7	NA	NA	NA	NA	NA	NA	NA	All: F-P; subcortical	NA	–
Vella and Mariani 1960 [111]	1	43, M	M +		+			PBA, disinhibition, anxiety, depression, apathy	NA	NA	–
Myrianthopoulos and Smith 1960 [78]	1	66, M	+	5 y	+	–	Memory, cognitive efficiency	Depression	P	NA	–
Parnitzke and Seidel 1961 [82]	2	P1: 16, M P2: 18, M	–	NA	–	–	–	Psychosis, depression	NA	NA	+
Alliez and Roger 1963 [4]	1	60, M	+	2 y	+	+	Cognitive efficiency	–	NA	EEG: widespread	–
Poppe and Tennstedt 1963 [87]	2	P1: 50, M P2: 65, F	All: +	NA	–	–	P1 and P2: memory, cognitive efficiency, orientation, language	P1 and P2: depression, psychosis	P1 and P2: widespread atrophy; AD + Pick’s	NA	–
Pisseri 1963 [86]	1	70, M	+	NA	–	–	Cognitive efficiency, language	Apathy, disinhibition, psychosis	NA	NA	+ (psychosis)
Beau 1964 [11]	1	22, M	+	NA	–	+	Language	Depression, psychosis	NA	EEG and CSF: negative	–
Von Matt 1964 [114]	1	57, F	+ M	6 mo	+	–	Memory, language, cognitive efficiency	Disinhibition, apathy	Widespread atrophy	NA	+ (psychosis)
de Morsier 1967 [32]	1	52, M	+	7 y	+	–	Attention, memory, language	Depression, anxiety, OCD, psychosis	F-T (T +); Pick’s; subcortical	NA	–
Caidas 1966 [20]	1	60, M	+ M	1 y	+	+	Cognitive efficiency, memory	Psychosis	F; sub-cortical	NA	–
Chateau et al. 1966 [24]	1	57, F	–	3 y	–		Memory, orientation	–	F-T atrophy	EEG: widespread abnormalities CFS: negative PEG: widespread atrophy	–
Boudouresques et al. 1967 [14]	1	54, M	+	40 m	+	+	Attention, memory	–	F-T (F +); sub-cortical	PEG: positive (F, subcortical) EEG and CSF: negative	+
Bonduelle 1975 [13]	2	P1: 65, M P2: 59, F	–	P1: 2 y P2: 3 y	All: +	All: +	P1: memory, cognitive efficiency P2: orientation, memory, calculation	P1: disinhibition P2: disinhibition, apathy	P1: F, sub-cortical P2: F-T (F > T), subcortical; AD	P1: EEG and CSF: negative; normal P2: EEG: widespread abnormalities; CSF: negative	
Dazzi and Finizio 1969 [30]	3	P1: NA, F P2: 53, M P3: 47, F	P1: + M	P1: NA P2: 1 y P3: 1 y	All: +	–	P1: cognitive efficiency P2: orientation, memory, attention, language, cognitive efficiency P3: –	P1: psychosis, disinhibition P2: – P3: PBA	NA	P1: NA P2: CT and CSF: negative P3: CT, EEG and CSF: negative	All: +
Minauf and Jellinger 1969 [74]	1	64, F	–	2 y	+	–	–	–	Pick’s; T	NA	–
Yuasa 1970 [122]	1	54, F	+	2 y	+	–	Cognitive efficiency	–	Pick’s F	EEG: widespread abnormalities PEG: F	–
Allen et al. 1971 [3]	1	56, M	+ M	13 mo	+		Memory, praxis	Psychosis	F-T; subcortical; CJD-like	EEG: CJD-like	–
Yvonneau et al. 1971 [123]	2	P1: 50, M P2: 65, M	All: +	NA	P1: +	–	P1 and P2: cognitive efficiency	P1: apathy, disinhibition P2: anxiety, disinhibition, psychosis	P1: widespread atrophy; subcortical	NA	+
Yase et al. 1972 [121]	1	32, M	+	11 y	+	+	–	Depression, psychosis, apathy, disinhibition	Widespread atrophy; sub-cortical; AD	EEG: P	–
Finlayson et al. 1973 [36]	2	P1: 60, M P2: 52, M	P1: + M P3: +	P1: 30 mo P2: 3 y	P1 and P2: +	–	P1: memory P2: cognitive efficiency, attention, praxis, visuo-spatial	P1: disinhibition P2: disinhibition, PBA	P1 and P2: F-T; sub-cortical	NA	+
Kaiya 1974 [57]	2	P1: 45, M P2: 61, M	All: +	P1: 20 mo P2: 3.5 y	+	–	P1 and P2: cognitive efficiency, orientation	P1 and P2: disinhibition	P1 and P2: widespread atrophy	NA	P1: + (intellectual disabilities)
La Maida et al. 1974 [60]	1	39, M	+	NA	–	+	Language, attention	Depression, Apathy	NA	NA	+ (depression)
Sherratt 1974 [97]	1	53, M	+	NA	–	–	Calculation Executive functioning	Disinhibition, anxiety	NA	EEG: CJD-like	–
Kurachi et al. 1979 [59]	1	48, M	+ M	15 m	+	–	Memory, cognitive efficiency	Disinhibition, anxiety	T (R > L); cerebellar; sub-cortical; Pick’s	NA	–
Pinsky et al. 1975 [84]	1	50, F	–	5 y	+	–	Memory, language, cognitive efficiency	Apathy	F-T (+ T); sub-cortical	NA	+
Hart et al. 1977 [52]	1	56, F	+ M	19 mo	–	–	Memory, language	Apathy	F-T	EEG: widespread	–
Ferguson and Boller 1977 [34]	2	P1: 68, M P2: 66, M	–	P1: 2 y P2: 3 y	+	–	P1: calculation, language P2: language	–	P1: F-P P2: NA	P1: EEG, CT, CSF: negative P2: EEG, CT: negative	–
Brion 1980 [15]	1	59, M	+	5 y	+	–	Cognitive efficiency, memory, executive functioning	Eating behavior, disinhibition, apathy	T (R > L)	PEG: F-T (R > L)	–
Burnstein 1981 [17]	1	55, F	–	5 y	–	–	Memory, language, visuo-spatial	Psychosis, depression, anxiety, apathy	F-T	CSF: normal EEG: T	+

*NPs*, neuropsychological; *EPD*, extra-pyramidal disorder; *ALS*, amyotrophic lateral sclerosis; *PBA*, pseudobulbar affect; *T*, temporal; *F*, frontal; *P*, parietal; *O*, occipital; *R*, right; *L*, left; *CC*, corpus callosum; *OFC*, orbitofrontal cortex; *OCD*, obsessive–compulsive disorder; *CJD*, Creutzfeldt–Jakob disease; *pts.*, patients; *NA*, not available; *y.*, years; *mo.*, months. In “Familial elements,” a simple “ + ” refers to the presence of ALS with or without cognitive/behavioral alterations within the family tree; familiarity for different neurological/psychiatric disorders has been specified. In “NPs onset,” “ + M” means that both NPs and motor symptoms were present at the onset

### Cognitive phenotyping

As to cognition, long-term memory deficits allegedly involving episodic, prospective, or autobiographical dimensions happened to be the most frequently reported (Table 1), this somehow anticipating the recently recognized notion of primary, medial-temporal amnesic features possibly characterizing ALS patients’ cognitive profile [83]. Moreover, as to medial-temporal lobe-rooted functions, a number of twentieth-century reports also described topographical disorientation both within and outside of dementing states (Table 1).

Among instrumental domains, deficits in calculation [12, 34], praxis [21, 73], and visuo-spatial skills [31, 73] were not infrequently reported over the twentieth century. In this respect, it is notable that such domains are currently regarded as either “ALS-nonspecific” or uncommonly occurring in ALS patients [26].

Deficits of non-instrumental functions, i.e., attention and executive functioning, also started to be more precisely reported (Table 1)—this being in agreement with the notion of frontal networks subserving such processes being altered in ALS [26].

### Language phenotyping

In respect to language, a number of contributions reported the occurrence of aphasic symptoms/syndromes both before and after the onset of ALS (Table 1). At times, language semiology in ALS patients was reported with a relatively high degree of details—e.g., predominant lexical-semantic deficits [116], or aphasic syndromes mostly affecting written language [40, 94].

Notably, Michaux et al. [73] described two ALS patients whose features were likely to meet current diagnostic criteria for semantic dementia (SD) [46], whereas Poppe and Tennstedt [87], within a series of patients with Pick’s disease co-occurring to either ALS or motor neuron signs, four patients presenting with either predominant lexical-semantic or morpho-syntactic involvement—i.e., resembling SD and progressive non-fluent aphasia (PNFA), respectively [46]. Both reports somehow anticipated not only the current notion of “PPA-ALS” (i.e., PPA patients with motor neuron dysfunctions) [102], but also that of both SD and PNFA possibly being co-morbid to ALS [99].

Furthermore, a number of the aforementioned reports not only described dysgraphic features [40,73, 87], which have been nowadays acknowledged as typical of ALS patients’ language profile [1], but also reading deficits—which have been thus far, by contrast, under-recognized. Taken together, such findings are strikingly consistent with the current knowledge on PPA-like language dysfunctions in ALS [85, 1, 96]—which, surprisingly, have been fully recognized as sufficient for a diagnosis of ALSci only in 2017 [99], with the first nosographic system rather focusing on dysexecutive features [100].

### Behavioral phenotyping

With respect to behavioral phenotyping, obsessive–compulsive spectrum symptoms (e.g., exaggerated hoarding) and disinhibited traits (e.g., personality changes and disrupts of social conduct) started to be increasingly reported (Table 1)—these nowadays representing recognized features of ALSbi/cbi and ALS-FTD, resembling those of bvFTD [90]. Moreover, the occurrence of psychotic features of a paranoid nature happened to be more frequently reported, both before [104] and after the onset of motor symptoms [38, 118]. It is noteworthy that the association between schizophrenia spectrum disorders and ALS/FTD is nowadays acknowledged, also on a genetic basis [126].

Furthermore, semeiotic description of dementing states co-morbid to ALS begun to be finer-grained when compared, for instance, to previous reports of “euphoric dementia” [37, 65]. A number of authors indeed started hinting at either a frontal-type [125] or a progressive aphasic dementia [116, 61, 73], in a way preceding the notion of bvFTD and PPA being the dementing phenotype co-occurring to ALS [99].

Moreover, different phenotypes of behavioral, dysexecutive-like features are distinguishable in certain twentieth-century reports, describing either manic-like, disinhibited [61] vs. predominant apathetic profiles [106], as well as the co-existence of both behavioral alterations [14]. The notion of different behavioral phenotypes within the ALS-FTD spectrum is indeed nowadays recognized [83].

Late-/early-onset, seemingly reactive depression, in the context of both spared and impaired neuropsychological functioning, were also described in a number of early-twentieth-century reports [39, 91, 35]. Interestingly, depressive symptoms of mixed psychogenic and organic etiology are now estimated as moderately-to-highly prevalent in ALS [53].

### The spectrum “read backwards”

Several contributions reported depressive symptoms, psychotic features, FTD-like behavioral changes as well as cognitive deficits/dementia preceding the onset of ALS (Table 1)—also by a timespan of years [109, 81, 6, 51]. Notably, such observations are consistent with the currently recognized possibility of neuropsychological symptoms appearing before motor signs in ALS-FTD spectrum disorders [75], as well as of motor neuron signs being likely to occur over the course of FTD (FTD-MND) [23]. In respect to the latter stance, the detection of pyramidal signs that however did not lead the authors to formulate a diagnosis of full-blown ALS within patients presumably presenting with FTD was not infrequently described [76, 112], this anticipating the abovementioned notion of the ALS-FTD spectrum possibly being “read backwards.” Indeed, nowadays, it is commonly recognized that patients diagnosed with bvFTD and PPA may show motor neuron signs [23].

### Bulbar signs as a risk factor

Twentieth-century authors also increasingly acknowledged that cognitive/behavioral disorders happen to be more prevalent when bulbar involvement occurs [12, 109, 125]—a relatively widespread notion nowadays [120]. Notably, reports of the association between bulbar-predominant ALS and FTD-spectrum disorders are also retrievable within the late nineteenth century [115].

## Histopathological records

Oppenheim and Siemerling [80] for the first time reported, in 1886, 5 patients with dementia and predominant-bulbar motor neuron signs whose autopsy revealed frontal and temporal atrophy. After a few years, also Sarbó [95] and Haenel [49] described relatively widespread, extra-motor cortical abnormalities in ALS patients showing dysexecutive, behavioral features.

However, neuropathological examinations of clinically diagnosed ALS patients showing cognitive/behavioral changes started to be more frequently reported and described with a higher degree of detail starting from the second decade of the twentieth century (Table 1). Such findings appear to be of even greater interest as often including, besides evidence on extra-motor involvement, histopathological verification of pyramidal system alterations (i.e., motor cortex and corticospinal tract) [72, 116, 61].

Pre-/orbital-/medial-frontal and temporal cortex involvement, at both macroscopic (atrophy) (Table 1) and microscopic levels (glial proliferation, astrocytosis, morphological neuronal alterations, and neuronal loss) [72, 116, 107, 110], were noted in a number of patients that nowadays would be likely to be classified as ALSci/bi/cbi, ALS-FTD, or FTD-MND, as well as fall under the relative spectrum of TDP-43 proteinopathies [18].

Moreover, sub-cortical white matter and diencephalic involvement (basal ganglia, thalamus, subthalamic nuclei) happened to be also reported (Table 1), consistently with the current notion of such structures possibly being affected by the spreading of both ALS and FTD pathology [18, 117]. Notably, Teichmann [103] and Kurachi et al. [59] also reported both macroscopic/microscopic cerebellar alterations within the post-mortem examination of ALS patients with neuropsychological involvement, this also being in line with the nowadays acknowledged possibility of the cerebellum being involved within the ALS-FTD spectrum [58].

Of interest, a number of reports allegedly succeeded in identifying the histological signature of Pick’s disease (Table 1), by also disentangling it from both Alzheimer’s disease (AD) [72, 94] pathology and senile-related physiological alterations or cerebrovascular lesions [31, 51]. By contrast, also the co-existence of Alzheimer’s and Pick’s disease pathology [87], as well as that of AD alone [125], happened to be reported—this being in line with the current knowledge of AD-like burden possibly being found at post-mortem examination of ALS cases presenting with neuropsychological changes [50].

Notes on lateralization and relative selectivity of damages can also be detected in a number of the aforementioned reports. For instance, Miskolczy and Csermely [76] envisaged a relevant anatomo-clinical correlation when describing an alleged FTD-MND patient with prominent language impairment whose post-mortem examination was suggestive of a left-greater-than-right frontotemporal cortex atrophy. Similarly, Kurachi et al. [59] described an ALS patient with prominent long-term memory impairment whose autopsy revealed a selective right-greater-than-left temporal pole atrophy—this possibly being the first description of ALS associated with the nowadays so-called right temporal variant FTD (rtvFTD) [27].

Overall, it is striking that, already in the first four decades of the twentieth century, the notion of a progressively spreading pathology beyond the motor cortex had been acknowledged as being the biological basis of neuropsychological changes in ALS [9, 48]—somehow anticipating current theories of sequential, corticofugal stages underlying both motor and cognitive/behavioral involvement within the ALS-FTD spectrum [93].

## In vivo cerebral evidence

Starting from the fourth decade of the twentieth century, several authors also reported in vivo evidence of neuroanatomofunctional changes in ALS patients showing neuropsychological impairments, albeit rarely and limitedly to a restricted range of instrumental examinations (Table 1)—i.e., pneumoencephalography, EEG, CSF analysis, and, starting from 1969, CT scans [30, 34].

In this respect, the report by Miskolczy and Csermely [76] is of great relevance, as being the first to concurrently described consistent in vivo and post-mortem findings in a probable PPA case who later developed motor neuron signs—i.e., a neuropathologically confirmed Pick’s disease patient whose EEG had showed alterations within the frontal and temporal lobes.

## Neuropsychological studies

As to the contribution of neuropsychology to the acknowledgment of the link between ALS and FTDs, the report by Lechélle et al. [61], Michaux et al. [73], Campanella and Bigi [21], and Boudouresques et al. [14] are of particular interest, as being the first to deliver psychometric evidence of cognitive dysfunctions—along with post-mortem and, at times, in vivo, neuroanatomofunctional correlations (Table 1).

Lechélle et al. [61] and Michaux et al. [73] described a series of ALS patient seemingly presenting with SD. A comprehensive battery of language tests indeed revealed a severe, progressive, and amodal impairment of the lexical-semantic component (with word frequency effects being also described), along with dyslexic (single-letter recognition deficits and predominantly phonological paralexias) and dysgraphic features (morpho-syntactic and phonological paragraphias). Notably, Michaux et al. [73] also described a preservation of object vs. action semantics—this possibly representing the first report of noun–verb dissociation within the ALS-FTD spectrum, a neurolinguistic phenomenon systematically documented within the last 30 years [85].

By contrast, Campanella and Bigi [21] and Boudouresques et al. [14] reported fine-grained semeiotic and psychometric descriptions of cognition and behavior in ALS patients with probable bvFTD—describing verbal inertia, apathy, anosodiaphoria, hypomanic features, and, at testing, predominant executive-attentive deficits, accompanied by possibly secondary dysfunctions of instrumental domains such as memory, praxis (including closing-in phenomena), visuo-spatial skills, and calculation.

Along with other reports alluding to psychometric testing in ALS patients [30], the abovementioned ones express an ante litteram need to objectively assess the cognitive/behavioral status of these patients. Notably, several ALS-specific psychometric screeners have been developed within the last decade, in order to provide cognitive/behavioral measures free from disease-related confounders (e.g., upper limb impairment during paper-and-pencil tasks or dysarthria within tasks requiring timed, verbal responses) [47].

## Familial incidence

Starting from the nineteenth century and more frequently in the twentieth century, a number of cases have been reported of familial and possibly genetic ALS patients presenting with cognitive/behavioral dysfunctions (Table 1), both across [123, 30] and within generations [21, 36].

A number of these reports specifically suggested an autosomal dominant transmission/a high genetic penetrance [30]. Relevantly, cognitive/behavioral phenotypes were often reported as similar within such familial/genetic cases—e.g., familial cases of progressive bulbar palsy with aphasic dementia [94], slowly progressing ALS with or without dementia [21], early-onset psychosis with dementia developing within the fifth/sixth age decades [123], or slowly progressive, juvenile-onset ALS with psychosis [77].

Of note, the report by [30], who described a series of familial, probable ALS-FTD patients within an Italian kindred was revisited and extended by Giannoccaro et al. [45], who followed a number of individuals belonging to the same family and performed genetic analyses in four of them, detecting mutations consistent with the current neurogenetics of ALS-FTD spectrum disorders, among which the *C9orf72* expansion. Giannoccaro et al. [45] concluded that the family described by [30] carried the *C9orf72* expansion.

Finally, it is noteworthy that, within twentieth-century case series of ALS with FTD-like involvement, a familiarity with other neuropsychiatric disorders (e.g., psychosis, mood disorders, and epilepsy) was noted (Table 1) this possibly representing an ante litteram recognition of the genetic association between the ALS-FTD spectrum and unrelated brain disorders, which is nowadays believed to be underpinned by the phenotypic heterogeneity yielded from *C9orf72* mutations [25]. Such evidence appear to be even more consistent when referring to psychotic disorders (Table 1), as well as in line with the current knowledge on schizophrenia spectrum disorders frequently occurring within the genealogical tree of ALS and FTD patients, possible due to *C9orf72*-related genotypes [70].

## Extra-pyramidal involvement

As early as 1963 [4], also the nowadays certified occurrence of extra-pyramidal systems possibly being involved in ALS with FTD-spectrum disorders [88] had been reported in Europe. It is of note that such cases were addressed as resembling the ALS-parkinsonism-dementia complex (ALS-PDC), identified as endemic in Guam and in the Kii peninsula starting from the 1950s (Supplementary Material 1).

For instance, Boudouresques et al. [14], who reported a French ALS patients with co-morbid frontal-like dementia who also showed parkinsonisms within the early stages of the disease. Similarly, La Maida et al. [60] described a patient whose onset symptoms included both pyramidal and extra-pyramidal involvement, as well as depressive and apathetic features.

## Conclusions

**Fig. 2:**
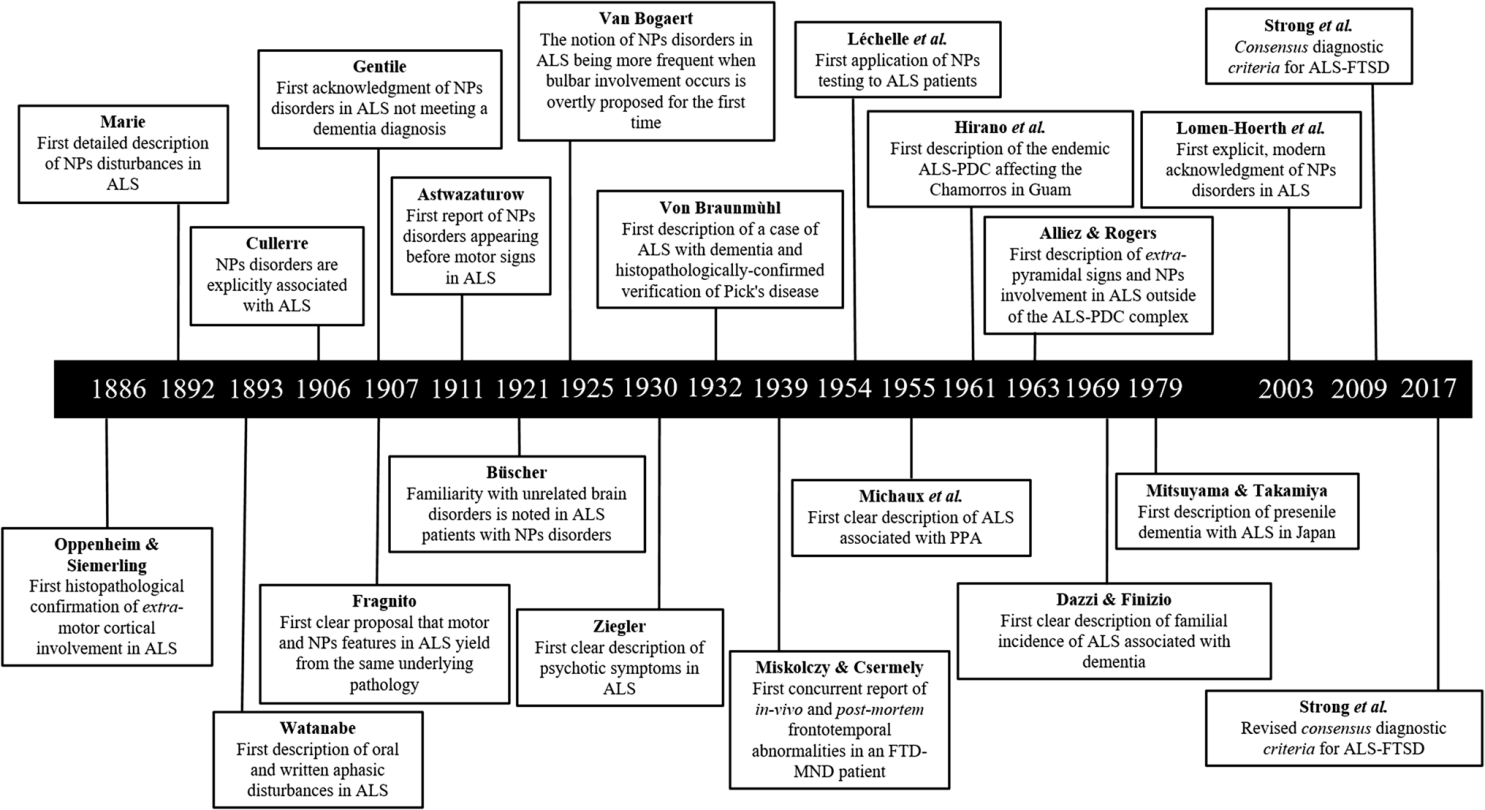
Timeline of historical milestones for the recognition of the association between ALS and FTD. Notes. ALS, amyotrophic lateral sclerosis; FTD, frontotemporal degeneration; NPs, neuropsychological; ALS-PDC, ALS-parkinsonism-dementia-complex; MND, motor neuron disease; FTSD, frontotemporal spectrum disorders; PPA, primary progressive aphasia

Within this historical review, strong evidence for the acknowledgment of extra-motor, frontotemporal-like cognitive/behavioral alterations in ALS dating back over 130 years ago is provided. Despite being flawed by the inherent lack of scientific progress nowadays achieved, these early reports outstandingly align with the current notion of ALS and FTDs being linked, not only at a phenotypic level but also from anatomofunctional, histopathological, and genetic points of view. It is indeed not incautious to state that several landmarks on the link between ALS and FTD had been reached way before the late 1990s of the twentieth century (Fig. 2). It has then to be noted that, between 1981 and the early 2000s, a number of reports can be traced that somehow paved the path to the full acknowledgment of the ALS-FTD spectrum occurred with the first, dedicated nosographic system by Strong et al. [100]—as indexed by a number reviews that elegantly summarized evidence at that time available, among the most remarkable being those by Strong et al. [101] and Neary et al. [79], with some other, relevant reports between 1981 and the early 2000s being also more recently brought to the light by Alberti et al. [2]. The present work also follows up to and completes the previous one by Bak and Hodges [7], who pioneeristically addressed certain of the historical records herewith described, and is complemented by an extremely recent, historical work by Carlos and Josephs [22] who focused on the centenary journey leading to the acknowledgment of the neuropathological basis of FTD-spectrum disorders.

Nineteenth- and twentieth-century authors also appear to urge modern neuroscientists to exert caution in addressing neurodegenerative conditions as discrete nosological entities, as well as to pay greater attention to semiology, since neuropsychology—a predominantly clinical discipline—should arguable be credited the most for sparking the fire that led to recognize that “the mind is *affected* in ALS.”

## Supplementary Information

Below is the link to the electronic supplementary material.

Supplementary file1 (DOCX 16 KB)

## Abbreviations

ALS: Amyotrophic lateral sclerosis
ALSbi: ALS with behavioral impairment
ALSci: ALS with cognitive impairment
ALScbi: ALS with cognitive and behavioral impairment
ALS-PDC: ALS-parkinsonism-dementia complex
ALS-FTD: ALS with frontotemporal dementia
FTD: Frontotemporal degeneration
bvFTD: Behavioral variant frontotemporal dementia
PNFA: Progressive non-fluent aphasia
PPA: Primary progressive aphasia
SD: Semantic dementia
