# Supplementary material for: Cognitive and behavioral involvement in ALS has been known for more than a century
Source: Neurol Sci. 2022 Sep 2;43(12):6741–60. doi: 10.1007/s10072-022-06340-0 (PMC9663353; doi:10.1007/s10072-022-06340-0)
Supplement: Supplementary file 1 — Supplementary file1 (DOCX 16 KB) [file 10072_2022_6340_MOESM1_ESM.docx]

**Supplementary Material 1.** The history of the ALS-PDC complex.

An highly incident “hereditary paralysis” among the indigenous Chamorro population of the Pacific Island of Guam, USA had been reported since the early ‘10s of the 20^th^ century, with an explicit reference to an ALS diagnosis first appearing on a death certificate in 1931 [1]. An ALS epidemic was indeed described in Guam starting from 1940 (with an incidence 50-100 times greater than that of the worldwide), prompting the National Institute of Neurological Diseases and Blindness of the National Institutes of Health to establish a field station on the island in 1956 [2]. A second entity, clinically characterized by a combination of motor neuron signs, *extra-*pyramidal symptomatology and dementia, *i.e.* the so-called “ALS-parkinsonism-dementia complex” (ALS-PDC) was discovered in equally high incidence on Guam and neighbouring islands in the Marianas chain a few years later.

Malamud *et al.* [3], in 1975, were the first to study two brain specimens from Guam ALS patients, one of whom also had dementia and parkinsonism, noting the unusual occurrence of Alzheimer’s neurofibrillary tangles. Elizan *et al.* [4] had instead the merit to define the cognitive profile of ALS-PDC patients as predominantly featured by a dysexecutive syndrome. From the ‘60’s, the clinical, neuropsychological, neuropathological, and genetic features of ALS-PDC started to be investigated in detail [1; 5; 6-12]. These works suggested that ALS and ALS-PDC among the Chamorros were variants of a single, endemic neurodegenerative process with a broad phenotypic *spectrum*, the etiology of which remained obscure after nearly 70 years of international investigations. Nowadays, is has been acknowledged that ALS-PDC is a multiple proteinopathy due to tau, α-synuclein, and TDP-43 aggregations presumably due to environmental factors (among which, toxins contained in the seed of locals cycad tree) [13]. In Japan, a variant of ALS-PDC has been also reported from the Kii Peninsula [14].

**References**

1. Hirano A, Malamud N, Kurland LT. Parkinsonism-dementia complex, an endemic disease on the island of Guam. - II. Pathological features. Brain. 1961; 84: 662-79.
2. Garruto R. M. et al. U.S. Department of Health and Human Services National Institutes of Health NIH Publication No. 83-2622, 1983.
3. Malamud N, Hirano A, Kurland LT. Pathoanatomic changes in amyotrophic lateral sclerosis on Guam. Special reference to the occurrence of neurofibrillary changes. Arch. Neurol. 1961; 45: 401-15.
4. Elizan TS, Hirano A, Abrams BM, Need RL, Van Nuis C, et al. The Amyothrophic Lateral Sclerosis and Parkinsonism-Dementia Complex on Guam: Neurological Re-evaluation. *Arch Neurol*. 1966; 14: 356-68.
5. Kaiya H. Zur Klinik und pathologischen Anatomie des Muskelatrophie-Parkinsonismus-Demenz-Syndroms. Arch. Psychiatr Nervenkrankd. 1974; 219: 13-27.
6. Lessell S. Hirano, A., Torres, J., & Kurland, L. T. Parkinsonism - dementia complex. Epidemiological considerations in the Chamorros of the Mariana Islands and California. Arch. Neurol. 1962; 7: 377–85.
7. Hirano A. Arumugasamy N, Zimmerman HM. Amyotrophic lateral sclerosis and parkinsonism - dementia complex on Guam. Further pathologic studies. Arch. Neurol. 1966; 15: 35-51.
8. Reed D, Plato C, Elizan T, Kurland LT. The amyotrophic lateral sclerosis/parkinsonism - dementia complex: ten-year follow-up on Guam. Part I. Epidemiologic studies. Am. J. Epidemiol. 1966; 83: 54-73.
9. Brody JA, Chen KM. Recent studies of amyotrophic lateral sclerosis and parkinsonism dementia on Guam. In: Proceedings of the Second Asian and Oceanian Congress of Neurology, Melbourne, Australia, 1967, May 1-5, E. Graeme Robertson, Editor, p. 331-34.
10. Plato CC, Garruto RM, Galasko D, Craig UK, Plato M, et al. Amyotrophic lateral sclerosis/parkinsonism - dementia complex of Guam. IV. Familial and genetic investigations. Am. J. Hum. Genet. 1967; 19: 617-32.
11. Eldridge R, Ryan E, Rosario J, Brody JA. Amyotrophic lateral sclerosis and parkinsonism - dementia in a migrant population from Guam. Neurology. 1969; 11: 1029-37.
12. Hudson AJ. Amyotrophic lateral sclerosis and its association with dementia. Parkinsonism and other neurological disorders: a review. Brain. 1981; 194, 217-47.
13. Spencer PS. Hypothesis: etiologic and molecular mechanistic leads for sporadic neurodegenerative diseases based on experience with Western Pacific ALS/PDC. Front. Neurol. 2019; 10: 754.
14. Mitsuyama Y. Presenile dementia with motor neuron disease in Japan: A new entity? Arch. Neurol. 1979; 36: 592-93.
